# Familiar Hypopigmentation Syndrome in Sheep Associated with Homozygous Deletion of the Entire Endothelin Type-B Receptor Gene

**DOI:** 10.1371/journal.pone.0053020

**Published:** 2012-12-31

**Authors:** Gesine Lühken, Katharina Fleck, Alfredo Pauciullo, Maike Huisinga, Georg Erhardt

**Affiliations:** 1 Department of Animal Breeding and Genetics, Justus-Liebig University of Giessen, Giessen, Germany; 2 Institute of Veterinary Pathology, Justus-Liebig University of Giessen, Giessen, Germany; Institut Jacques Monod, France

## Abstract

In humans, rodents and horses, pigmentary anomalies in combination with other disorders, notably intestinal aganglionosis, are associated with variants of the endothelin type-B receptor gene (*EDNRB*). In an inbred Cameroon sheep flock, five white lambs with light blue eyes were sired from the same ram and died within a few hours up to a few days after birth, some of them with signs of intestinal obstruction. The aim of this study was to investigate if the observed hypopigmentation and a possible lethal condition were associated with a molecular change at the ovine *EDNRB* locus, and to check if such a genetic alteration also occurs in other Cameroon sheep flocks. Sequence analysis revealed a deletion of about 110 kb on sheep chromosome 10, comprising the entire *EDNRB* gene, on both chromosomes in the two available hypopigmented lambs and on a single chromosome in the two dams and three other unaffected relatives. This micro-chromosomal deletion was also confirmed by quantitative real-time PCR and by fluorescence in situ hybridization. Genotyping of a total of 127 Cameroon sheep in 7 other flocks by duplex PCR did not identify additional carriers of the deletion. Although both hypopigmented lambs available for post-mortem examination had a considerably dilated cecum and remaining meconium, histopathological examination of intestinal samples showed morphologically normal ganglion cells in appropriate number and distribution. This is to our knowledge the first description of an *ENDRB* gene deletion and associated clinical signs in a mammalian species different from humans and rodents. In humans and rats it is postulated that the variable presence and severity of intestinal aganglionosis and other features in individuals with *EDNRB* deletion is due to a variable genetic background and multiple gene interactions. Therefore the here analyzed sheep are a valuable animal model to test these hypotheses in another species.

## Introduction

Lethal white foal syndrome (LWFS, OMIA #000629-9796) is an autosomal-recessively inherited condition of newborn foals born to American Paint Horse parents of the overo coat-pattern linage [1], [2]. The foals are totally or almost totally white and affected with intestinal aganglionosis [3]–[5], leading to a functional obstruction (megacolon) and death. A mutation in the endothelin type-B receptor gene (*EDNRB*) was found to be the cause for LWFS in American [6], [7] and Australian [8] Paint horses. Clues to this molecular basis came from man and rodents, where disorders are also observed that associate abnormal skin coloration and pigmentation patterns or white coat spotting, respectively, and intestinal aganglionosis.

The inheritable human Waardenburg syndrome (WS) is characterized by the association of pigmentation abnormalities, including depigmented patches of the skin and hair, vivid blue eyes or heterochromia irides, and sensoneural hearing loss. The association of these disorders results from an abnormal proliferation, survival, migration, or differentiation of neural crest-derived melanocytes [9]. Four subtypes of WS were defined on the basis of the presence or absence of additional clinical signs [10]. Type I WS (WS1) and WS2 are characterized by great variability of clinical signs, however both cover heterogeneous collection of melanocyte defects and they can be distinguished by the further presence of dystopia canthorum in WS1. WS with musculoskeletal abnormalities of the upper limbs and dystopia canthorum has been called Klein-Waardenburg syndrome or WS3. WS4, also known as Hirschsprung disease (HD) type II, or Shah-Waardenburg syndrome (OMIM #277580), is defined by the association with HD [11]–[14]. HD or aganglionic megacolon is a congenital defect characterized by an absence of neural crest-derived intramural ganglia along varying lengths of the colon [15]. Single nucleotide substitutions and deletions in the gene encoding the endothelin type-B receptor are associated with a prominent portion of WS4 cases and a small percentage of WS2 cases, respectively [9]. A white coat colour in combination with intestinal aganglionosis is also observed in mice with targeted disruption or natural (piebald-lethal) mutations of the *EDNRB* locus [16], and in the spotting lethal rat, carrying an interstitial deletion of the *EDNRB* gene [17].

In an inbred flock of Cameroon sheep, five totally or almost totally white-coated lambs with light blue eyes were born. All died within few hours up to few days after birth, and signs of intestinal obstruction were noticed in some cases.

The aim of this study was to investigate if the observed lethal hypopigmentation syndrome was associated with genetic variation at the *EDNRB* locus, and to check if such a possible genetic variant would also be found in other Cameroon sheep flocks.

## Results

### Evidence for Obstruction but not for Aganglionosis in Hypopigmented Lambs

**Figure 1:**
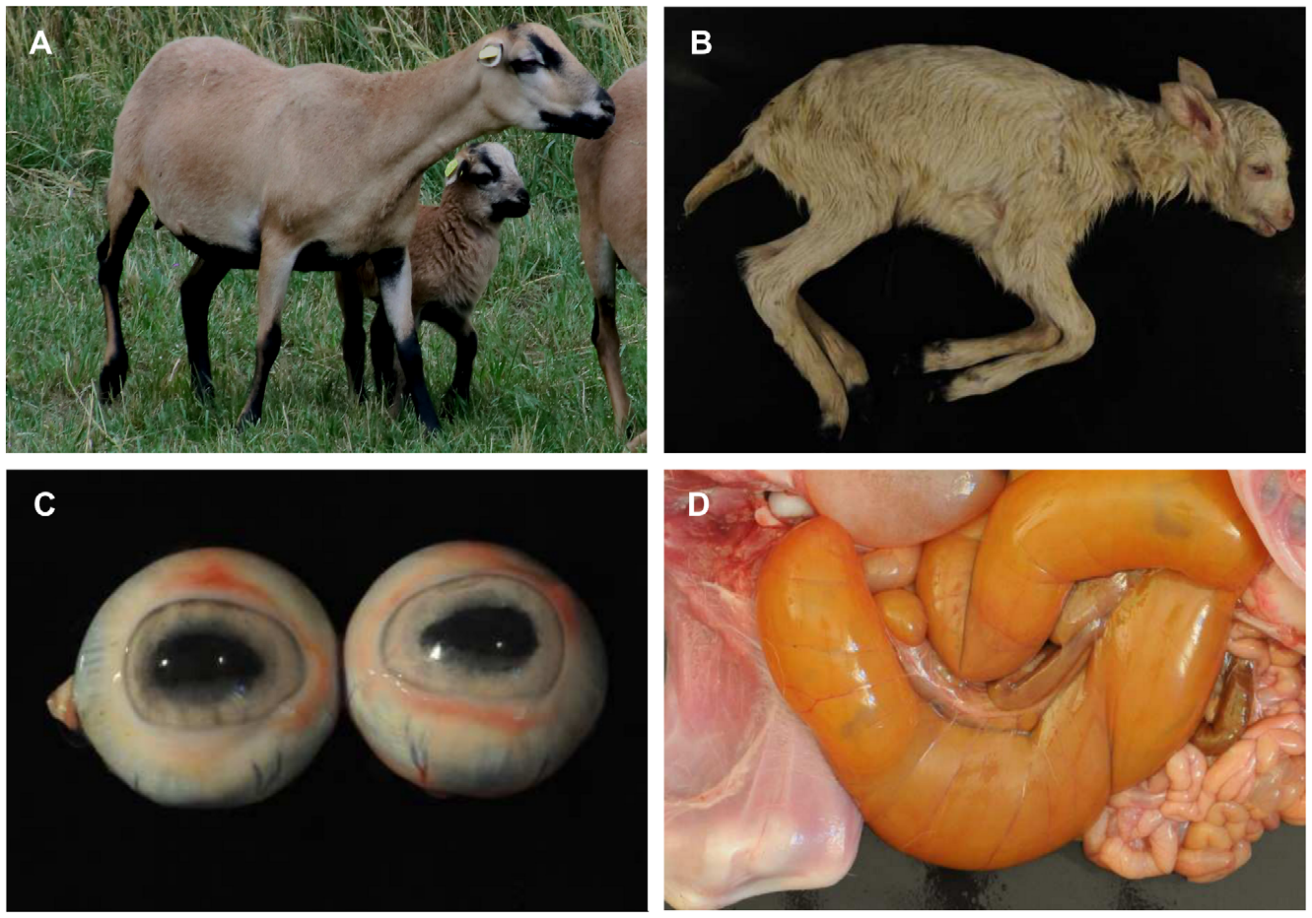
Phenotypically normal and hypopigmented Cameroon sheep. (A) Brown Cameroon sheep ewe and lamb. (B) Hypopigmented Cameroon sheep lamb, with light blue irides (C), and dilated cecum (D).

Two of the hypopigmented lambs were available for post-mortem examination. Instead of the common brown phenotype of Cameroon sheep (figure 1A), one of the lambs was totally white, whereas the other was also white but had pigmented distal limb ends including the claws and black marks in a small perianal area (figure 1B). In both lambs, the irides were coloured light blue (figure 1C), instead of dark brown as it is usual for Cameroon sheep. Both showed a considerably dilated cecum (figure 1D) and remaining meconium. Histopathological examination of intestinal samples of both lambs revealed a normal number and distribution of ganglion cells which showed a homogenous faint labelling for synaptophysin by immunohistological investigation. By bacteriological examination of samples from both lambs *E. coli* could be cultured from the intestine, mesenterial lymph nodes, liver, spleen, kidneys and lung. The body of one of the lambs was rather fresh at post-mortem examination (necropsy 24 hours post mortem). Therefore, the evidence of *E. coli* in many organs was interpreted as a final sepsis. The organs of the other lamb were already autolytic when necropsy was performed. Hence the detection of *E. coli* was without informative value.

### Amplification of *EDNRB* Sequences Failed Exclusively in Hypopigmented Lambs

The coding regions including flanking parts of ovine *EDNRB* were amplified in order to sequence and to characterize the gene in affected lambs as well as in unaffected sheep. In DNA samples from the two hypopigmented lambs, none of the primer pairs hybridizing to *EDNRB* sequences (table 1) was able to amplify a PCR product, whereas amplification was successful with all primer pairs in DNA samples from all flock mates (n = 27) and Cameroon sheep from other flocks (n = 5). Coding and flanking *EDNRB* sequences generated by sequence analysis of PCR fragments were submitted to GenBank (accession number JQ937242).

**Table 1:** Details on PCR amplification of ovine *EDNRB* fragments.

amplified *EDNRB* region*	fragment size (bp)	annealing temperature (°C)	sequences of forward and reverse primers (5′-3′)	
I† –1	694	60.0	CTGCTGCGCTTCAGGATAG CCTGCAAAGACTTTCCCATC	
1– II –2– III –3	825	56.0	TGCAGATGATTTTCAGAGGAG TGAGAATCAGGGAATTCTTGG	
3– IV –4	366	52.0	GAAGATTATTCCTTGATGAGCATTT CAGACTAAGAAAAAGGAATTATGCTCT	
4 –V –5– VI –6	1036	52.0	CAAATGCCACTGACTTTTTGT CAAGGGAAAATTATAAAACAGTTGA	
6– VII	784	52.9	TGAGCAAGGAGGGTTGTGAT CTGTCTGATTCTCCCTCCTGA	

* amplified fragments are not overlapping; Roman numerals = exons, Arabic numerals = introns;

† partially amplified exon (66 bp of 5′ end missing).

### Ancestors of Hypopigmented Lambs had Lower Relative *EDNRB* Copy Numbers than Unrelated Sheep

**Figure 2:**
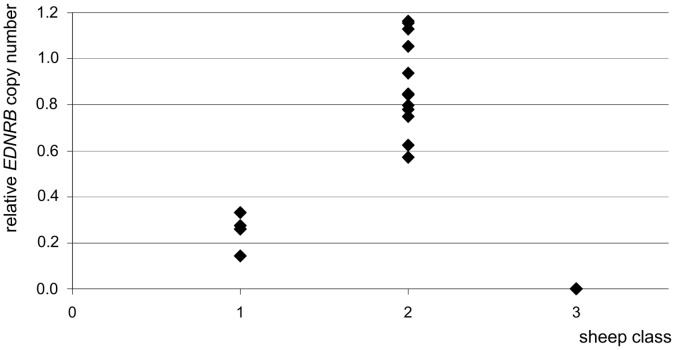
Relative *EDNRB* copy numbers resulting from real-time PCR. Values of single sheep are shown in classes: 1, dams and (great-) granddam of hypopigmented lambs (n = 4); 2, unrelated sheep (n = 12); 3, hypopigmented lambs (n = 2).

A quantitative real-time PCR was conducted on fragments of the *EDNRB* gene and a control gene for the purpose of estimating potential variation in the *EDNRB* gene copy number. Mean values of repeated measurements for relative *EDNRB* gene copy numbers of three dams and one granddam and great-granddam of hypopigmented lambs, of 12 unrelated sheep (Cameroon sheep from same flock and other flocks), and of two hypopigmented lambs are shown in figure 2. In DNA samples from ancestors of hypopigmented lambs, the relative *EDNRB* copy number was significantly (p<0.001) lower (mean value: 0.252±0.079) than in DNA samples from 12 unrelated sheep (mean value: 0.887±0.202). No amplification was observed in DNA samples from hypopigmented lambs.

### Micro-chromosomal Deletion Responsible for *EDNRB* Gene Lack

**Figure 3:**
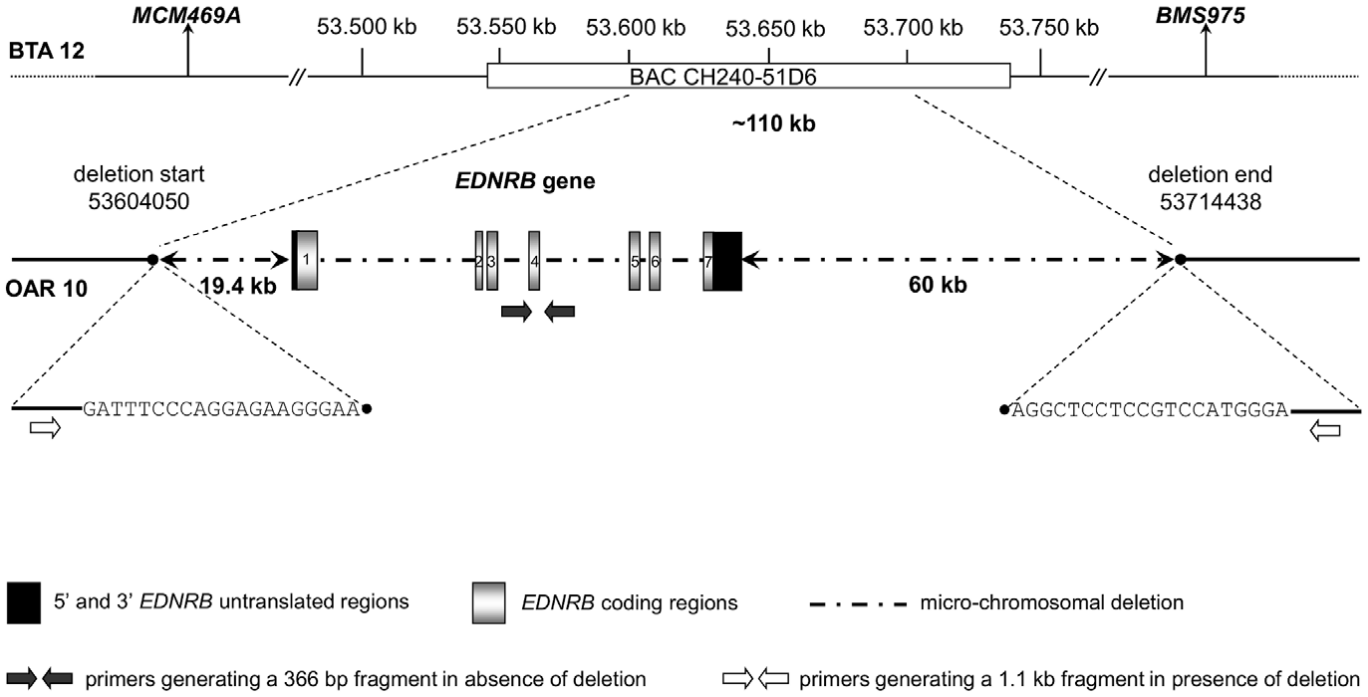
Schematic representation of a micro-chromosomal deletion on OAR 10 including the *EDNRB* locus. Indicated positions refer to the bovine genomic sequence NC007310.4 (BTA 12, Btau4.0).

In order to identify the starting and the end point of the deleted sequence, a set of reference microsatellites and DNA fragments was amplified. PCR amplification was successful in DNA samples from the two hypopigmented lambs and unrelated sheep for the microsatellite *MCM469A* and 5 further fragments located upstream of *EDNRB*. The same for the microsatellite *BMS975* and 5 fragments downstream of *EDNRB* (figure 3). No amplification exclusively in DNA samples from the two hypopigmented lambs was obtained for 7 assessed primer pairs located 18.7 kb upstream and nearer to *EDNRB*, as well as 58.9 kb downstream and nearer to *EDNRB*, respectively. Finally, a primer pair located 19.4 kb upstream and 60 kb downstream to *EDNRB*, respectively, generated a 1.1 kb fragment (figure 3) in samples from the hypopigmented lambs, their dams and some other related sheep only. Comparison of this fragment with the available homologous bovine sequence (NC007310.4) revealed a gap of approximately 110,000 base pairs, spanning from position 53,604,050 to position 53,714,438 (figure 3). For this DNA region all nucleotide positions indicated in this paper refer to the bovine genomic sequence, because currently it is the only available without gaps.

Generated sequences of chromosome regions upstream and downstream of *EDNRB* were submitted to GenBank (accession numbers JQ937243, JQ959537 and JQ959538).

**Figure 4:**
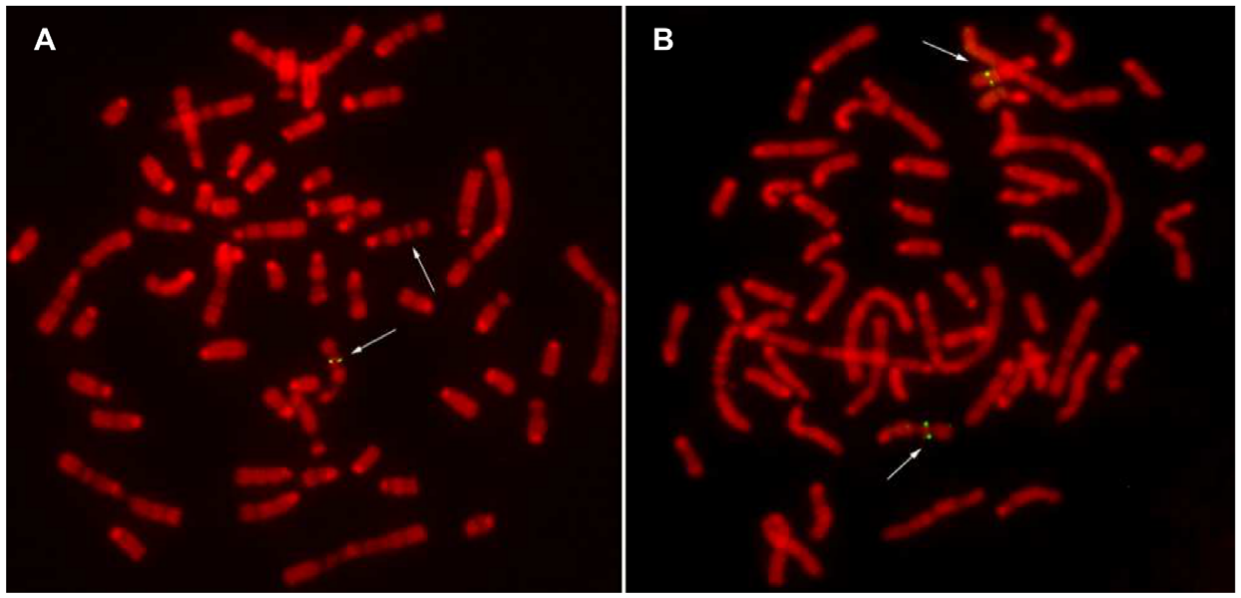
Fluorescence in situ hybridization of an *EDNRB*-spanning bovine BAC clone on metaphase chromosomes. Results from a sheep related to hypopigmented lambs (A), and from an unrelated sheep (B). Arrows indicate positions of (expected) hybridization signals.

Using a BAC clone which spans the entire *EDNRB* gene including the flanking regions, a fluorescence in situ hybridization (FISH) based method was developed to confirm the gene deletion also from a cytogenetic point of view. Three sheep were analyzed. Two of them were related to the hypopigmented lambs and heterozygous carriers of the 110 kb deletion, as confirmed by real-time PCR and duplex PCR (described below). RBPI-banding identified the location of the *EDNRB* gene on chromosome 10q22. FISH analysis showed only two symmetrical spots on the metaphases of the carriers of *EDNRB* gene deletion, whereas no signal was detected on the other chromosome 10 (figure 4A). The normal sample showed four distinct signals on the two homologous chromosomes (figure 4B). This finding confirmed the micro-chromosomal deletion to be located on band q22 of the ovine chromosome 10. The analyzed sheep related with the hypopigmented lambs were heterozygous carriers of such mutational event.

### Testing for *EDNRB* Deletion by Duplex PCR Identified Further Deletion Carriers in the Affected Flock but not in Unrelated Cameroon Sheep

**Figure 5:**
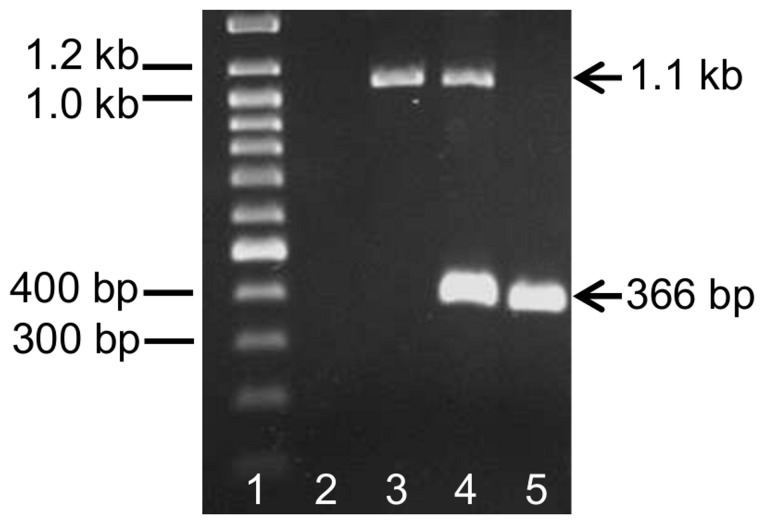
Agarose gel electrophoresis of DNA fragments resulting from duplex PCR. Lane 3: sample from sheep homozygous for the deletion (*EDNRB −/−*), lane 4: sample from sheep heterozygous for the deletion (*EDNRB +/−*), lane 5: sample from sheep without the deletion (*EDNRB +/+*); lane 1: DNA size marker (Gene Ruler 100 bp Plus DNA Ladder, Fermentas, St. Leon-Rot, Germany); lane 2: no template control.

A duplex PCR was set up as diagnostic test for quick identification of deletion carriers. As confirmed with sequenced samples, the established duplex PCR reliably differentiates sheep missing the *EDNRB* gene on a single (EDNRB +/−) or both (EDNRB −/−) chromosomes, and unaffected sheep (EDNRB +/+). As demonstrated in figure 5, a single 366 bp fragment occurs in sheep without the deletion (lane 5), whereas this fragment together with a 1.1 kb fragment indicate heterozygous carriers of the deletion (lane 4, sample from a dam of a hypopigmented lamb). Only a single 1.1 kb fragment is amplified in samples from sheep homozygous for the deletion (lane 3, hypopigmented lamb). Positions of the primers for the 366 bp and 1.1 kb fragments are also indicated in figure 3.

All available sheep of the affected flock (n = 27) were analyzed by duplex PCR. In total 13 heterozygous carriers of the deletion were identified, including the 3 dams and the granddam and great-granddam of the hypopigmented lambs already analyzed by sequencing and quantitative real-time PCR. All 13 EDNRB +/− sheep were progeny or ancestors of the sire of hypopigmented lambs.

A total of 127 Cameroon sheep (126 brown and 10 pied) from 7 different flocks were tested for the *EDNRB* deletion by duplex PCR. However, no additional carriers were identified.

## Discussion

In our study, we were able to show that a hypopigmentation syndrome occurring in a family of Cameroon sheep was associated with a homozygous 110 kb interstitial deletion on chromosome 10, including the entire *EDNRB* gene. The *EDNRB* gene has previously been assigned to the sheep chromosome 10 in the region q22 relative to the standard G-banded chromosome ideogram [18], whereas the gene maps to bovine chromosome 12q22 [19] and to chromosome 13q22 in humans [20].

We could also verify the expected recessive inheritance of the phenotype, as normal brown dams of affected lambs were heterozygous carriers of the deletion. Although the two investigated lambs showed signs of obstruction, unaltered ganglion cells were demonstrated by histological and immunohistological studies in all samples taken at different sites of the intestine. Therefore the association of intestinal aganglionosis with a deletion of the *EDNRB* gene as described in humans [21]–[24] and rodents [16], [17], [25] could not be confirmed in sheep. However, if the aganglionosis was restricted to a very short intestinal segment it may have been overseen. Reliable diagnosis of ultra-short HD in humans requires an enzyme-histochemical acetylcholinesterase reaction of serially native sections of distal rectal mucosa [26]. On the other hand, ultra-short aganglionosis develops with milder clinical sings of constipation and in a higher age compared to aganglionoses of longer segments [26], [27]. Therefore it is unlikely that an ultra-short aganglionosis would have caused the death of the two lambs, which died shortly after birth.

In any case, it remains open if the observed hypopigmentation was associated with a lethal condition (in this study undiscovered), or if the lambs finally died in consequence of an infection as frequently seen in newborn lambs (e.g., E. coli sepsis [28]).

From horse and rodents it is known that the coexistence of hypopigmentation and intestinal aganglionosis does not necessarily depend on the degree of molecular changes at the *EDNRB* locus. In the American and Australian Paint horses, a substitution of only two nucleotides in the coding *EDNRB* sequence results in the substitution of a single amino acid (Ile118Lys) and is associated with the lethal white foal syndrome in animals homozygous for this genetic variation [6]–[8]. In turn, a deletion of 301 bp, encompassing the distal half of the first exon and the proximal part of the adjacent intron of *EDNRB*, is underlying a similar phenotype in the spotting lethal rat [17], [25]. This deletion results in the absence of a functional receptor protein [25], [29]. The total whiteness and megacolon associated with a naturally occurring deletion of the complete *EDNRB* locus in homozygous piebald-lethal mice is quite similar to the phenotype caused by a targeted disruption of the gene in *EDNRB* knock-out mice [16].

Similar to the horse, in the majority of *EDNRB*-associated human WS4 cases (mostly homozygous) missense mutations are observed, caused by single nucleotide substitutions. A few truncating mutations and deletions were also characterized (reviewed by [9], [22]). Most human *EDNRB* variations are private and their overall transmission is complex, but it can be considered that individuals homozygous for these mutations have a high probability of developing severe phenotypes, while heterozygous patients may present one ore more features of the disease with low or incomplete penetrance. The observation that the same variants in different families results in different phenotypes argue for an influence of the genetic background [9].

Even among the few human cases of a 13q deletion (including the entire *EDNRB* locus) associated with WS4 [22], only one case with the full WS4 phenotype (discoloured irides, HD and sensorineural hearing loss) was reported [21]. Moreover, in this case the deletion was defined by high resolution Comparative Genomic Hybridization (CGH), but it was hypothesized by the authors that the features were secondary to a mutation in the other allele. Pigmentary anomalies alone or together with hearing loss, but without HD were observed in several of the human 13q deletion syndrome cases [22], [30]–[32]. Similar to the affected lambs in our study, in a single human patient, megacolon was shown, but ganglion cells were identified on biopsies taken at different parts of the rectum and thus, HD could not be confirmed [22]. However, to our knowledge, all described human *EDNRB* deletion cases were heterozygous, therefore the variable penetrance of the phenotype could be due to *EDNRB* mutations on the other chromosome, as already postulated [32].

Even more comparable with the hypopigmented Cameroon sheep lambs in our study was the situation investigated in different inbred rat lines with the spotting lethal rat mutation [33]. Although all three analyzed rat lines were homozygous for the same null mutation of the *EDNRB* gene, they differed in occurrence and severity of clinical signs. Most strikingly, all animals suffered from intestinal aganglionosis in two lines, while in more than a half of the rats from the third line, no aganglionosis was observed. There was also a significant difference in the extent of aganglionosis between the three lines, from reaching from above the cecum to only a very short segment near the anus. The genetic backgrounds also affected the severity of pigmentation abnormalities. As there was no correlation between the extent of aganglionosis and pigmentation loss, the authors concluded that the modifier genes of these two phenotypes were different in the analyzed rat line [33].

We set up a quantitative real-time PCR to verify the hypothesis of a causative recessive gene deletion before seeking to identify its detailed extent by sequencing flanking chromosome regions. As expected, a significantly lower mean *EDNRB* copy number was observed in samples from dams and a granddam and great-granddam (all later proven to be heterozygous) of the hypopigmented lambs than in unrelated sheep (all later proven not to carry the deletion). However, real-time results from single animals were quite variable, not exactly showing the expected ratio of 2∶1 (without vs. single chromosome *EDNRB* deletion). Thus, a duplex PCR was also established as diagnostic tool for screening for *EDNRB* deletion carriers. We included also pied sheep in the sampling as it was possible that - similar to human cases – a variable penetrance of WS4-like features could lead to pigmentation abnormalities also in heterozygous sheep. However, besides the 13 sheep in the Cameroon flock where the hypopigmented lambs occurred, no additional *EDNRB* deletion carriers were identified. All of the hypopigmented lambs were sired by the same ram. Therefore it is very likely that he or one of his ancestors was the founder of the identified micro-chromosomal deletion and that there was no spread into other Cameroon sheep flocks.

A FISH-based approach using a BAC probe was also useful for a simple and rapid detection of the chromosomal deletion including the *EDNRB* gene. To our knowledge, a deletion of the entire *EDRNB* locus has not been described in farm animals before. However, both in sheep and in river buffalo, *EDNRB* is located in a chromosomal region which was indicated as fragile site [34], [35]. Therefore, it is not unlikely that the identified micro-chromosomal deletion might have been generated by an abnormal recombination event or by a chromosomal breakage. Even though in this study we have identified only a single affected family, the identified micro-chromosomal deletion opens further interesting prospective of investigation also in the field of FS in sheep.

## Materials and Methods

### Lethal Hypopigmentation Syndrome in a Cameroon Sheep Flock

**Figure 6:**
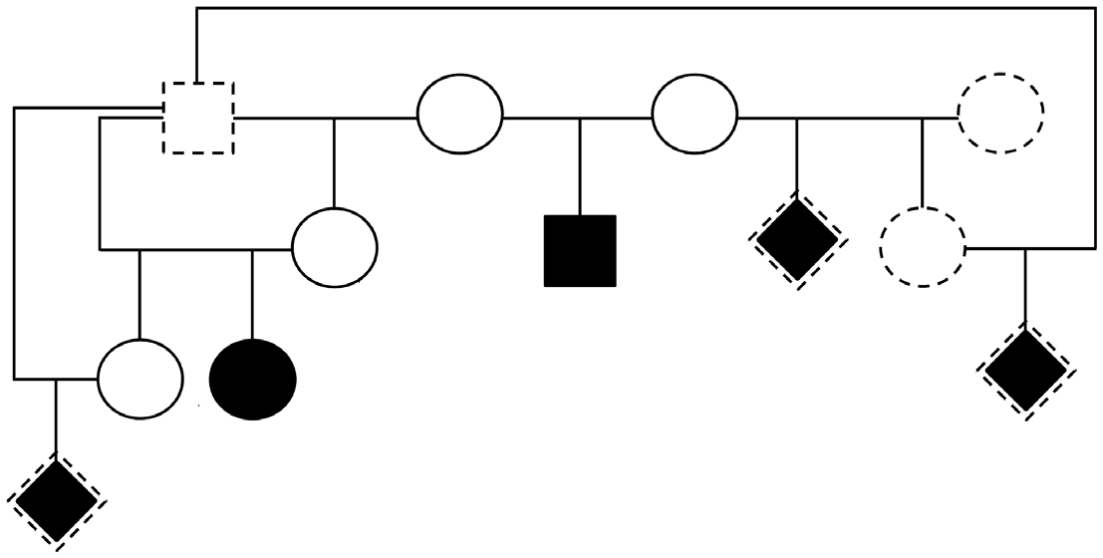
Pedigree of hypopigmented lambs. Square: male; circle: female; rhomb: sex unknown; empty symbol: phenotypically normal; filled symbol: affected; symbol with dashed margin: not sampled.

In a small flock of Cameroon sheep, a single brown ram sired all lambs for at least 3 consecutive years and was also mated to his female progeny. These ewes were also brown besides one animal with a single white spot in the hip area. The owner reported that among their progeny had been five totally or almost totally white-coated lambs with light blue eyes, which all died within few hours up to few days after birth. The lambs seemed to be healthy at birth, but the owner did not remember that they defecated. All of these lambs became depressed and then recumbent before they died. An abdominal distension was noted by a veterinary at least in one of the lambs; however, the medical histories of the cases were not documented in detail. Furthermore, pedigree information is incomplete, and due to inbreeding, difficult to describe as a whole. Nevertheless, all available pedigree information was in accordance with genotyping results for 9 microsatellites (*BM8125*, *ILSTS11*, *ILSTS28*, *INRA063*, *MAF33*, *MAF70*, *OARFCB128*, *OARJMP58*, *OARVH72*), which were analyzed in DNA samples from the two affected lambs and all sampled flock members. Not all affected lambs and related sheep were available for examination and sampling, including three of the hypopigmented lambs, two of their dams, and the sire of all lambs. Figure 6 shows the essential pedigree of the five hypopigmented lambs as well as the availability of samples from these sheep.

### Post-mortem Examination

A routine post-mortem examination of two of the hypopigmented lambs was performed 3 days and 1 day after they died. Samples of the small and large intestine were collected (e.g. duodenum, jejunum, ileum, cecum, colon, rectum) and fixed in 10% formalin. They were routinely processed for histology, and embedded in paraffin. 4 µm sections were cut from paraffin blocks and stained with haematoxylin and eosin. Additional sections were used for the immunohistochemical staining with synaptophysin (clone SY 38, DakoCytomation, Hamburg, Germany). Detailed information is given elsewhere [36], [37]. A routine bacteriological and virological examination was done with samples from liver, spleen, kidney, lung, intestine, and intestinal lymph nodes.

### Sample Collection and DNA Isolation

Tongue tissue was taken from the two hypopigmented lambs during *post-mortem* examination and used for isolation of genomic DNA with a commercial kit (NucleoSpin Tissue Kit, Macherey Nagel GmbH & Co. KG, Düren, Germany). Blood samples were taken by punction of the *Vena jugularis*. Blood was sampled from all existing flock mates (n = 27), including 3 dams and a granddam and great-granddam of hypopigmented lambs (figure 6) using EDTA-monovettes. Further blood samples for cytogenetic investigation were collected from 3 sheep using Na-heparine monovettes. Additional blood samples (n = 127) were collected in 7 other Cameroon sheep flocks using EDTA-monovettes. Of these sheep, 117 were brown haired and 10 were pied (brown with some white parts of the body). There was no incidence for any pedigree links between the affected flock and these additionally sampled flocks. Genomic DNA was isolated from blood by high salt-method [38].

Ethical Statement for the use of animal material: The study was done according to the German Animal Welfare Act. On the basis of article 8 (7) 2a of this law, no notification of or approval by the Animal Protection Unit of the Regional Council of Gießen, Germany, was necessary for this study.

### PCR Amplification and Sequencing of EDNRB Fragments

For amplification of the ovine *EDNRB* coding region, PCR primers were designed using the bovine genomic sequence (GenBank NC_007310.4, Btau4.0). Along the *EDNRB* gene five fragments covered the coding area as well as flanking noncoding parts. PCR amplifications were carried out according to manufacturer’s standard protocol for Promega Go Taq polymerase. Amplified regions, fragment sizes, annealing temperatures and primer sequences are given in table 1. Resulting PCR products were sequenced on both strands with the PCR primers using Big Dye Terminator chemistry and the ABI 3130 Genetic Analyzer (Applied Biosystems, Foster City, CA, USA). Each product was sequenced in samples from 10 sheep. 5 samples were from relatives of the affected lambs and 5 samples were from unrelated Cameroon sheep. Alignment and analysis of sequences from the different samples was done with the software ChromasPro version 1.33 (Technelysium Pty Ltd, Tewantin, Australia).

### Quantitative Real-time PCR Amplification

The relative *EDNRB* copy number was assayed by comparing quantitative real-time PCR results of an *EDNRB* fragment and of a control fragment from the SLAIN motif family member 1 gene (*SLAIN1*), being located about 150 kb upstream of *EDNRB*. For this purpose, a 147-bp fragment from exon IV of *EDNRB* (forward primer 5′-GAAGATTATTCCTTGATGAGCATTT-3′ and reverse primer 5′-CGGCAGGCAGAAATAGAAAC-3′) and a 151-bp fragment from exon III of *SLAIN1* (forward primer 5′-GCGAAGTCTCTTCCCTTCAA-3′ and reverse primer 5′-TTTTAAAAGCACATTTGGAAATACA-3′) were amplified and verified by sequencing as described above. For real-time PCR, 0.2 µM of each primer was used with the Absolute qPCR SYBR Green Mix (Thermo Scientific Abgene, Schwerte, Germany) as recommended by the manufacturer. The assay was performed in a Corbett Rotor Gene RG 3000 cycler (Corbett Life Science, Mortlake, Australia) with 15 min at 95°C, followed by 40 cycles of 95°C for 15 sec, 54°C for 15 sec and 72°C for 15 sec. Finally, a melting curve analysis was done on reaction products to confirm specific amplification. A standard curve for the calculation of amplification efficiency and template concentration was constructed with a DNA sample from a sheep unrelated with hypopigmented lambs and serial 1∶1 dilutions (50%, 25%, 12.5%). All samples were tested as triplicates and no template controls were included in the assay. The quantification was carried out by Rotor-Gene Real-Time Analysis Software 6.0 (Corbett Research 2004). The amounts of *EDNRB* and *SLAIN1* templates were quantified and the ratio between the two was calculated for all samples. Two-sided t-test for unpaired samples was used to test the means for significant differences between groups. Homoscedasticity was ascertained using Levene-test. The statistical evaluation was carried out with the software PASW Statistics 18 (IBM, New York, USA).

### PCR Amplification and Sequencing of OAR 10 Sequences Upstream and Downstream of the EDNRB Locus

The presence of sequences flanking the *EDNRB* locus was in a first step tested by amplification of the two microsatellite markers *MCM469A* and *BMS975*, located on OAR 10 in a distance of about 1 Mb upstream and downstream of *EDNRB*, respectively. Information on primer sequences, PCR conditions and references for *MCM469A* and *BMS975* are given elsewhere (http://rubens.its.unimelb.edu.au/~jillm/jill.htm). This was followed by a step-by-step amplification of OAR 10 sequences located between *MCM469A* and the first exon of *EDNRB*, and between *BMS975* and the last exon of *EDNRB*. The distance of these fragments to *EDNRB*, fragment sizes, annealing temperatures and primer sequences are provided as supplementary table S1. All resulting PCR fragments were sequenced as described above.

### Duplex PCR

A duplex PCR was established for reliable and efficient identification of carriers of the *EDNRB* deletion. 0.5 µM of each of the forward and the reverse primer 5′-GAAGATTATTCCTTGATGAGCATTT-3′ and 5′-CAGACTAAGAAAAAGGAATTATGCTCT-3′ were used to amplify a 366 bp *EDNRB* fragment, ranging from intron 3 to intron 4 of the gene. In the same reaction, 0.16 µM of each of the forward and the reverse primer 5′-CACACAGAGAATCAGAAACAGAGAA-3′ and 5′-ATTGCTAGCTTAATTTCCTTTCTTTG- 3′ were included, being located about 19.4 kb upstream and 60 kb downstream of *EDNRB*, respectively, therefore spanning about 110 kb of OAR 10. PCR reactions were performed with the Qiagen Multiplex PCR Kit (Qiagen, Hilden, Germany) according to the manufactures recommendations and with the following temperature profile: 15 min at 95°C, followed by 30 cycles of 30 sec at 94°C, 90 sec at 55°C and 90 sec at 72°C, and a final elongation for 10 min at 72°C. After electrophoresis in a 1.5% agarose gel, PCR reaction products were visualized by ethidium bromide staining.

### Fluorescent in situ Hybridization (FISH)

Peripheral blood cell cultures from 3 sheep were treated for late-incorporation of BrdU (15 µg/ml) to obtain R-banding preparations for chromosome analysis by FISH. Hoechst 33258 (30 µg/ml) was simultaneously added to BrdU 6 h before harvesting to enhance the R-banding patterns.

NCBI clone finder resource (http://www.ncbi.nlm.nih.gov/clone/) was used to choose a bovine BAC clone including the *EDNRB* gene and flanking regions. The clone CH240-51D6 mapping in bovine 12q22 was purchased from the BAC/PAC collection belonging to Children’s Hospital Oakland Research Institute (CHORI, Oakland, CA, USA). DNA isolation from the BAC was carried out according to the alkaline lysis miniprep protocol suggested by CHORI. Before labeling, the DNA was tested via PCR for *EDNRB* locus (amplicon 366 bp long) using the same primers as described in the previous paragraph and standard PCR conditions.

Approximately 0.5 µg of the BAC DNA was labeled with biotin-16dUTP using standard nick translation kit (Roche, Mannheim, Germany) and used for RPBI-FISH (R-banding and propidium iodide staining) according to [39].

The hybridization mixture containing the BAC probe was applied on the slides and covered with 24×24 mm coverslips. The slides were incubated in a moist chamber at 37°C for 3 days. After hybridization and slide washing, detection steps were carried out with fluorescein isothiocyanate (FITC)-avidin (Vector Laboratories, CA, USA) and anti-avidin antibody (Vector Laboratories, CA, USA). Slides were mounted with antifade/propidium iodide (3 µg/ml).

Slides were observed at 100× magnification with a DMRA fluorescence microscope (Leica, Wetzlar, Germany) equipped with DAPI, FITC and Texas Red (TXRD) specific filters. Digital images were captured and analyzed with Leica Q4000 software.

## Supporting Information

Table S1 Details on PCR amplification of fragments upstream and downstream of ovine *EDNRB.*
(DOC)
