# Supplementary material for: Familiar Hypopigmentation Syndrome in Sheep Associated with Homozygous Deletion of the Entire Endothelin Type-B Receptor Gene
Source: PLoS One. 2012 Dec 31;7(12):e53020. doi: 10.1371/journal.pone.0053020 (PMC3534075; doi:10.1371/journal.pone.0053020)
Supplement: Table S1 — Details on PCR amplification of fragments upstream and downstream of ovine EDNRB. (DOC) [file pone.0053020.s001.doc]

Table S1: Details on PCR amplification of fragments upstream and downstream of ovine *EDNRB*

| Distance^*^ between fragment and *EDNRB* | fragment size (bp) | annealing temperature (°C) | sequences of forward and reverse primers (5’-3’) |
| --- | --- | --- | --- |
| 150 kb upstream (*SLAIN1*) | 409 | 59 | GCGAAGTCTCTTCCCTTCAA TTTTAAAAGCACATTTGGAAATACA |
| 42.742-43.080 bp upstream | 338 | 58 | TGGCCTTAGGGGTAAGAATG CAGAACTCATCTGGGGAGGA |
| 28.655-29.057 bp upstream | 402 | 60 | TCAAACAGGCTTTTCCTGGT TTCAGCAAAACAGCAATACTCC |
| 20.104-20.599 bp upstream | 495 | 55 | ACTGCAAAGAATGAGAAAGCA TTGGTCTTCTATGAATCCAATAGGT |
| 18.925-19.426 bp upstream | 501 | 55 | GATGACTGAATAAAGCAGCCCTA CACACAGAGAATCAGAAACAGAGAA |
| 18.322-18.732 bp upstream | 410 | 58 | CCTGAAATCAGGGGTCATGT CGCAAAGGGTCAGACACAAC |
| 17.220-17.646 bp upstream | 426 | 60 | GATTGGTGGGAGAGGTGCTA TGGATGCACTGAGGCAGATA |
| 16.350-16.807 bp upstream | 457 | 60 | GGCCATCTTGGTGACAGTTT TGTGGTGCCACGTTACAGAT |
| 13.129-13.533 bp upstream | 404 | 60 | TTTGGTGGTCCTCTTTCACC AAGGATCACATTGTTCCCTGA |
| 213-607 bp upstream | 394 | 62 | AAAGCTTTGAGCTCCTGGTG TATCCTGGGGCTTCAGTTTG |
| 3095-3498 bp downstream | 403 | 61 | GGCTGGTGAGCCTTACATTC TGAATTCAGGAGCTATGTGAGAA |
| 58512-58.977 bp downstream | 465 | 60 | TGCCCTGATGTCCTGTGTAA TTGCAAAGAGCAAACAATGC |
| 59.862-59.987 bp downstream | 125 | 60 | ATTGCTAGCTTAATTTCCTTTCTTTG AAAAAGGCATATATTGGAGACAAGA |
| 61.505-61.997 bp downstream | 246 | 60 | TCCCTTCCCTCATTGAACTG TTCAGAGAAACCTGGTGTGC |
| 64.278-64.686 bp downstream | 408 | 55 | ATAGTGGGGGCAATGGATTT TCAAAGTCCCATTTCTAGAACATTAC |
| 69.504-69.911 bp downstream | 407 | 55 | TTTCTGGTGCCTCAGTCAAG CTACCCACTCCTACCCACCA |
| 83.908-84.334 bp downstream | 426 | 57 | CACAGGCCTGGGTATCATTC TAGGTCCCTGAGATGGATCG |

^*^refers to bovine genomic sequence NC_007310.4
